# Health system strategies and responses to the effects of Climate Change in Sub-Saharan Africa: A scoping review

**DOI:** 10.1371/journal.pone.0349448

**Published:** 2026-06-17

**Authors:** Chancy Skenard Chimatiro, Solange Mianda, Precious Hajison, Martina Lembani

**Affiliations:** 1 School of Public Health, University of the Western Cape, Cape Town, South Africa; 2 Machinga District Health Office, Liwonde, Machinga, Malawi; 3 PreLuHa Consultancy, Zomba, Malawi; World Health Organization, CONGO

## Abstract

**Background:**

Climate change is now regarded as a global health challenge of the 21st century, posing a negative health risk to the population. Sub-Saharan Africa is disproportionately affected more than any other region worldwide. Mapping strategies and responses in Sub-Saharan Africa to address the impact of climate change on health systems is a starting point for understanding evidence-based decision-making and informing best practices.

**Methods:**

We conducted a scoping review to identify health systems strategies and responses to the effects of climate change in Sub-Saharan Africa. Electronic database searches were conducted on African Index Medicus, PubMed, CINAHL, and Scopus. Covidence software was used to remove duplicates, blind study selection, and data extraction. We included peer-reviewed articles (original quantitative and qualitative studies, mixed methods studies, reviews, editorials, and commentaries) published between 2011 and 2025. All book chapters and grey literature publications (dissertations, conference proceedings, abstracts, and reports) that primarily focus on climate change strategies and responses without effects on health systems were excluded. The results were analysed using descriptive thematic analysis.

**Results:**

Out of 11459 articles, 8 studies met our inclusion criteria. Most studies provided strategies and responses centered on service delivery, health workforce, health information, leadership and governance, with few on health financing and medicinal products. No study was identified that had outlined strategies and responses across all of the six World Health Organisation building blocks for the health systems. Implementation challenges identified include inadequate funding, lack of knowledge among health workers on climate change and health, inadequate surveillance and reporting structures, and low prioritization of climate change activities among health workers.

**Conclusions:**

This scoping review has identified some health system strategies and responses to the effects of climate change within the Sub-Saharan African region. The review has revealed that existing strategies and responses are fragmented and hindered by some implementation challenges. As climate change continues to pose health threats to the global population, urgent and effective interventions are required to minimize its impacts. It is essential to understand the unique vulnerabilities of the health systems, particularly those in the Sub-Saharan African region. The time is now to develop strategies and responses that can improve and strengthen health systems as it protects health of the population from the effects of climate change.

## Introduction

It is a well-known fact that health systems have been destabilized by the effects of climate change worldwide. The World Health Organisation (WHO) defines a health system as all organizations, people, and actions whose primary intent is to promote, restore, or maintain health [1]. It is composed of the six building blocks, including service delivery, health financing, medical products, vaccines and technologies, human resources, health information, and leadership and governance, that interact as a unit to promote health outcomes [1]. Health systems are responsible for a wide range of healthcare services from community to health facility levels to promote good health for the population [2,3]. These responsibilities include health promotion, prevention, curative, and rehabilitation healthcare services, which require health systems to be resilient to the external forces, including climate change [3,4].

Resilience is the ability and agility of a system to change and flex according to circumstances, and continue to function under stress, while changing [5]. In this case, the WHO described health system resilience as the ability of all actors and their functions to collectively mitigate, prepare, respond, and recover from disruptive events while maintaining the provision of healthcare services to the population [6]. Resilient health systems are supported and characterized by their capacity to anticipate, respond to, cope with, recover from, and adapt to climate-related stress and shocks to sustainably improve population health, despite unstable conditions [7]. However, the majority of health systems have been impacted by the effects of climate change, which poses public health risks to the population globally [8,9].

Climate change (CC) is defined as the state of the climate identified statistically by changes in the mean and/or the variability of its properties that persist for an extended period, typically a decade or longer [10]. Its causes are directly related to human activities, such as deforestation and combustion of fossil and biomass fuels [11], resulting in greenhouse gas emissions that accumulate in the atmosphere [12,13]. It is manifested through an increase in average global temperature, directly linked to more frequent and prolonged extreme weather events [14]. CC has negatively affected health systems as it is associated with several public health risks. It expands geographical range for disease vectors, increases air and water pollution, exacerbates outbreaks of infectious diseases and extended drought periods, leading to food shortage and malnutrition [15–17]. Furthermore, CC-induced floods have been reported to destroy health system infrastructure, limiting access to healthcare services worldwide [18,19]. The most vulnerable to the effects of CC are health systems with less resilience due to low socioeconomic status, which cannot adapt and effectively respond to changes [20].

Geographically, CC is disproportionately impacting Sub-Saharan Africa (SSA) more than any other region globally. According to the World Bank, 42 out of 48 SSA countries are among the high-risk countries facing negative health outcomes resulting from the effects of CC [21]. The region is characterized by a high poverty level, socio-economic dependence, and increased burden of infectious diseases, lowering its ability to respond to the effects of CC [22,23]. Moreover, the SSA is continuously experiencing the occurrence of the CC extreme weather events, such as increased heat, droughts, changes in rainfall patterns, and floods [24]. The occurrence of extreme weather events in the SSA is associated with negative social and health outcomes, including infrastructure damage, food shortages, increased multiplication of infectious diseases, and loss of properties and lives [25,26]. So far, the region is considered a CC hotspot with strong physical and ecological effects on health, due to its poor socioeconomic status [27]. Furthermore, the majority of health systems in this region are usually overwhelmed by the increase in healthcare service demand immediately after the occurrence of CC extreme weather events [28]. Despite SSA being disproportionately affected, global evidence indicates that fewer efforts are made to develop strategies and responses that can enhance health systems to be resilient to CC events within the region [29,30]. As a result, the majority of health systems within the region are unable to respond effectively.

Therefore, this scoping review was conducted to map the available strategies and responses to the effects of CC within the SSA. Specifically, the review identified strategies and responses used to manage the effects of CC on health systems and identified the challenges faced when implementing the strategies and responses. The guiding research question for this scoping review was, “What are the health systems strategies and responses to the effects of climate change in Sub-Saharan Africa?” The research question was developed considering the Population, Concept, and Context (PCC) framework [31]. The lessons drawn from this review will increase the clarion call on health systems to invest more in CC response and adaptation plans within SSA countries [32].

## Materials and methods

We conducted this scoping review in line with the methodological framework by Arksey & O’Malley updated by Peters [33,34], based on the following five stages: (i) research question identification, (ii) review of relevant studies, (iii) study selection, (iv) data charting, and (v) summarizing and reporting results. The Preferred Reporting Items for Systematic Reviews and Meta-Analyses extension for Scoping Reviews (PRISMA-ScR) guidelines [35,36] (Supplementary Table 1) were followed to guide the reporting of the results for this scoping review. In addition, the protocol for this scoping review was registered with the Open Science Framework and has been published in a peer-reviewed journal [37].

### Eligibility criteria

This scoping review included peer-reviewed articles (original quantitative and qualitative studies, mixed-methods studies, reviews, editorials, and commentaries) published between January 2011 and December 2025 to capture and map a comprehensive overview of available information and opinions on strategies and responses. Furthermore, the period January 2011 to January 2025 was chosen as it spans a decade, which aligns with the CC definition [10]. All book chapters and grey literature (dissertations, conference proceedings, abstracts, and reports) publications primarily focusing on CC strategies and responses without effects on health systems were excluded.

### Identification of relevant studies and search strategy

A comprehensive literature search of studies on strategies and responses to the effects of CC on health systems in SSA was conducted in several databases. The databases include Africa Index Medicus, PubMed/Medline, CINAHL, and Scopus. In addition, we searched gray literature on relevant websites, including those of the World Health Organization, Clim-Health Africa Resources, and Alliance for Transformative Action on Climate and Health Repositories. Full-text articles were obtained using search strings containing keywords using the ‘AND’ and ‘OR’, Boolean operators (Supplementary Table 2). The search words were developed in line with the PCC framework. The search word strings were further modified to suit each database after the PubMed/MEDLINE search. The search was conducted multiple times from November 1, 2024, and was completed on January 31, 2025.

### Study selection

All searched articles were downloaded and exported into the Covidence software. The search yielded 11,459 articles, of which 2058 articles were identified as duplicates, which were removed internally by Covidence software. After title and abstract screening, 34 studies were retrieved for full-text article review. A further 26 were removed because they did not meet the eligibility criteria after full article review by the two reviewers. Eight studies were included for data extraction.

**Fig 1:**
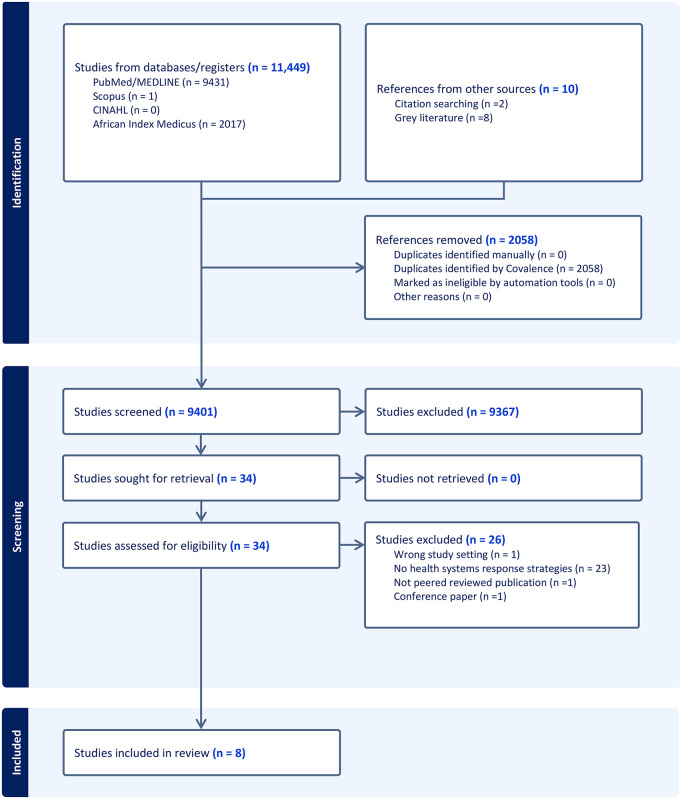
The PRISMA flow diagram indicating number of articles identified, included, and excluded.

Two stages were implemented during the study selection process. The first stage involved determining study eligibility based on the inclusion criteria by reviewing the study titles and abstracts. This stage was conducted blindly by the two reviewers, who had an agreement level of over 95% on the articles to be reviewed in full using the Covidence software. All screening disagreements between the two reviewers were resolved by inviting the third reviewer. In the second stage, two reviewers independently read the identified articles to determine the availability of the desired outcomes. The reviewers agreed 100% after reading the full articles; as such, no third reviewer was required. Data extraction quality checks and supervision were conducted by the fourth reviewer. All these stages, including the development of the PRISMA flow diagram, were internally done using the Covidence software (Fig 1).

### Data extraction

Two reviewers independently extracted data from the included studies. Pilot testing was performed before the extraction to ensure inter-rater agreement among the reviewers. Data were charted into Microsoft Excel, which included the following categories: author (s), year of publication, location, study type, study title, climate change response strategies, and implementation challenges. The reviewers cross-checked one another after the extraction process for consistency of the results. Any disagreements that arose during the extraction process were discussed and resolved using consensus by the two authors. This was performed independently by two reviewers who compared the results and reached consensus to ensure that all required information was captured correctly.

### Collating, summarizing, and reporting results

The extracted data were then synthesized and analysed, guided by the review’s overarching main aim and objectives. Specifically, we conducted a descriptive thematic analysis to conceptualize characteristics of the included studies and then identified the response strategies and implementation challenges. The results are presented in both tabular and narrative form. Information on the tabular form included author(s), year, location, study title, study design, climate change response strategies, and implementation challenges (Supplementry Table 3). We grouped narrative information into themes in line with the six WHO building blocks for health systems for an in-depth understanding of how each building block is being addressed in response to CC. The alignment of the six WHO building blocks was done with the help of academic health systems experts.

### Ethics

The scoping review does not involve the use of human subjects; as such, no consent or ethics clearance was required. However, as part of a larger study, the ethics clearance was approved by the Biomedical Research Ethics Committee (BMREC) of the University of the Western Cape, reference No. **BM24/9/6**.

## Results

### Appraisal of selected studies

The Mixed Method Appraisal Tool (MMAT) version 2018 was used to evaluate the quality of the selected articles [38]. During MMAT appraisal, two independent reviewers conducted the quality assessments. Each reviewer evaluated each criterion by selecting one of the three options: yes, no, or unsure, and ultimately offered a descriptive summary of the study. The third reviewer was invited to resolve any disagreements that emerged between the first two reviewers.

### Characteristics of the selected studies

Characteristics of included studies are displayed in tabular form (Supplementary Table 3). Out of the eight included studies, two were review studies [39,40], two quantitative studies [41,42], two qualitative studies [43,44], one systematic review [45], and one mixed-methods study [46]. The included studies were from Ghana (n = 2), Senegal (n = 1), Ethiopia (n = 1), Tanzania (n = 1), South Africa (n = 1), and two multi-country studies. One of the multi-country studies was conducted in Ghana, Nigeria, South Africa, Namibia, Ethiopia, and Kenya. Another multi-country study was included, although one of the countries (Egypt) was not from Sub-Sahara Africa region out of the 18 included countries. This study was included as it presented study findings from each country separately, and we did not include findings from Egypt. The included findings were from Botswana, Eritrea, Gambia, Ghana, Guinea-Bissau, Kenya, Lesotho, Malawi, Mauritius, Namibia, Nigeria, Rwanda, Sierra Leone, South Africa, Sudan, Swaziland, Seychelles, Uganda, Zambia, and Zimbabwe. All the included articles were published within the past eight years.

### Health system building block strategies for mitigating the impact of climate change

#### Service delivery.

This review identified response strategies that health systems use to ensure the continuous delivery of services during extreme weather events. These response strategies include the establishment of community groups to provide health services within the communities [43–45] and the provision of care outside the healthcare system through mobile and outreach clinics [41,43]. Health systems further task-shifted community health workers by training and allowing them to provide basic healthcare services in their catchment areas [44,45], which non-technical officers are trained to provide some of the health services usually provided by technical officers. Use of home-based care providers [43,44] is another response strategy used to ensure the continuation of service delivery. Distribution of mosquito nets, vector spraying, and promotion of water, sanitation, and hygiene practices immediately after a climate change extreme event [40,42,45] is another response strategy used to reduce the spread of infectious diseases. In addition, some health systems enhance service delivery through service integration by combining several professional disciplines [43,45] when providing healthcare services.

#### Health workforce.

Health systems are responding to the effects of climate change by building the capacity of health workers through training to improve their climate change response knowledge. Building the capacity of health workers through short-term training on climate change responses has been identified as a health system response strategy [41,43–46]. Moreover, the review identified that some health systems recruit additional permanent health workers [43] to the existing health workforce. Some health systems recruit surge health workers working on a contract basis [42] to cope with the high demand for health services during extreme weather events.

#### Health information.

Health systems deploy some response strategies to ensure adequate generation and dissemination of health information in response to climate change. The use of early warning systems [42,45,46], regular data collection, and the application of data for decision-making [40,45] are among the strategies reported to enhance the health system’s response to climate change events. Some health systems create public awareness through radio and television programs, in addition to community health promotion messages on water, sanitation, and hygiene practices [40,42] as response strategies to reduce the spread of infectious diseases. Strengthening research on climate change and health is another response strategy employed [40,45,46] to gather new evidence for decision-making and to inform policy best practices.

#### Medical products, vaccines, and technologies.

To ensure the availability of drugs, health systems use a contingency plan to identify drug suppliers [43] who distribute drugs during emergencies. Furthermore, health systems are adapting new techniques for the storage of drugs [41,44] by modifying architectural designs that suit a changing climate and adapting to extreme weather events and other environmental stressors. Health system infrastructures are equipped with alternative electricity backup systems (solar power supply) [40,41,44] to maintain the cold chain for proper storage of drugs during extreme weather events like extensive heat.

#### Health financing.

Little has been reported on health financing, as only two articles were obtained. One of the response strategies identified is that health systems lobby for additional funds from implementing partners and international donors [44,45] during the occurrence of extreme weather events such as floods. Some health systems redirect resources planned for other activities [43] towards climate change response as a strategy being used to boost health financing.

#### Leadership and governance.

This review identified that some health systems have developed national adaptation plans, and others are mainstreaming response strategies into existing health policies [39,40] to guide their response to climate change extreme events. Some health systems are also advocating a multi-sectoral approach [45,46] to form a risk response team as a strategy. Other health systems have developed climate change-specific health policies [40,46] to guide their operations and responses. Building resilient infrastructures [40,44] is another governance response strategy that other health systems are using. Furthermore, health systems are using carbon-free energy sources, such as solar power systems [40,41], as a response strategy to reduce the release of carbon dioxide into the atmosphere.

#### Implementation challenges identified.

The main challenge identified for the effective implementation of health system response strategies was inadequate funding to support climate change-related activities [42,45,46]. Insufficient knowledge on the effects of climate change on health among health workers and shortage of human resources [39,42,44,45], inadequate surveillance systems and weak reporting structures, such as health information on climate change risks [40,43,44], low prioritization of climate change activities among health workers, and increased workload during climate change events [39,44] have also been identified to hurt the implementation of strategies and responses. Furthermore, the lack of an exhaustive mapping of areas with a high climate vulnerability index [40] and poor planning for climate change events [41] were among the challenges identified in this review.

## Discussion

This review mapped evidence on health systems strategies and responses to the effects of CC, as well as implementation-related challenges, in the SSA region. Based on our objectives, this review found existing evidence on the strategies and responses, as well as implementation challenges. Overall, the study has mapped strategies and responses across each of the six WHO building blocks of the health system. The majority of strategies and responses identified focus on governance and leadership, human resources, health information management, and service delivery, with little evidence on health financing and essential medical products, vaccines, and technologies. The review identified studies from different methodological approaches, including reviews, qualitative, quantitative, and mixed-method study designs. In addition, we identified available evidence on implementation challenges faced by the health systems that need to be addressed by all actors for effective CC responses across all six WHO building blocks of the health systems.

In this review, no study was identified outlining strategies and responses that comprehensively address all six WHO building blocks of the health system. All identified strategies and responses addressed specific building blocks of the health system, disregarding their interactions. This reveals a lack of a holistic approach when developing strategies and responses within SSA, which limits their effectiveness. In this case, unattended health system building blocks may indirectly reduce the effectiveness of strategies and responses, resulting in undesirable outcomes for the targeted building block. Similar findings were reported in another study on systems thinking in practice, the current status of the six WHO building blocks for health system strengthening in three districts of Zambia, which found that building block-specific weaknesses had a cross-cutting effect on other building blocks [47]. The isolated strategies and responses for the selected building blocks can lead to system failure, potentially increasing the vulnerability of health systems to CC [48]. Hence, the weakness in one of the six WHO building blocks of the health systems affects others [49], which is a vital component of a functioning system.

It is interesting to note that some of the identified health system strategies and responses include the use of health information. Elsewhere, the use of health information technologies has been reported to strengthen health systems’ ability to predict and project the occurrence of CC-related extreme events, which prompt preparedness for effective response [50]. In addition, another study reported that health information technologies, such as early warning systems, provide opportunities to detect potential health risks and aid early response preparedness before health hazards occur [50]. It further reported that if used properly, early warning systems may reduce financial costs and protect vulnerable health systems from catastrophic financial spending [50]. Furthermore, Rahimi-Ardabili et al. (2022), in their review on digital health for climate change mitigation and response, reported similar findings [51]. In addition, it is reported that integrating climate data into national health information systems aids better planning in case of future events [52].

On the other hand, the study has revealed that some countries are incorporating strategies and responses into existing national health policies and adaptation plans to further strengthen their health systems. This provides proof that some countries within the SSA region are taking the effects of CC seriously and are ready to find better solutions to build resilient health systems. In this regard, it is important to take note of the suggested guidelines and tools by Rios and Thomson (2024) when incorporating CC strategies and responses into existing national health policies and adaptation plans [53]. We assume that, if carefully incorporated, it will help health systems in the SSA region to be more resilient and be able to effectively respond to CC events.

This scoping review has revealed a few strategies and responses to strengthen medical products, vaccines, and technologies, and health financing mechanisms. This being the case, we assume that fewer decisions are made to enhance the health system’s capacity regarding medical products, vaccines, and technologies, and health financing when responding to CC events. Moreover, insufficient information on health finance implies that the majority of health systems within the SSA region depend on donated funds from partners, more especially from international donors, or redirect already available funds towards CC activities during the occurrence of extreme events [54]. Although this approach facilitates the continuous provision of healthcare services, it may result in partial implementation of CC-related activities if donors are reluctant or if funds to be redirected are not available. This might affect the health systems’ ability to cope with and recover from CC events. Similarly, other studies reported that little attention had been given to essential medical products, vaccines, and technologies, and health financing to enhance CC response [55–57]. It is important that CC funds are ring-fenced in national budgets for health systems to aid prompt response.

This scoping review has revealed some evidence gaps for the effective implementation of the strategies and responses. Gaps in funding, insufficient knowledge among health workers on CC and health, and inadequate surveillance systems are some of the challenges faced. Overall, the identified gaps may hinder the ability of the health systems to timely recover from CC-related stresses and shocks. Similar findings were also reported in another scoping review on climate change and health systems [58]. Interestingly, another study reported on the need to intensify green climate change insurance, and strengthen current bilateral and multilateral programmes as an alternative way to enhance CC funding [59]. Apparently, health workers are increasingly aware of the rising CC-health risks but require formal training and better infrastructure that can overcome CC stresses [60]. The current review has an implication to prompt future research to explore the capacity of the health systems to conduct CC vulnerable assessments. Furthermore, this review may prompt future studies to understand mechanisms that can improve the CC health financing system, health system resilience, and surveillance systems.

## Limitations

This scoping review has some limitations. The findings presented here have not captured the global health system’s CC strategies and responses, which limits the generalizability of the findings. Another notable limitation is the exclusion of book chapters and grey literature publications (dissertations, conference proceedings, abstracts, and reports), which primarily focus on CC strategies and responses without effects on health systems, which limits the comprehensiveness and depth of the identified evidence. This is so because the primary focus of this study was to map the strategies and responses aimed at strengthening the health system to the effects of CC. However, these limitations were addressed through a comprehensive search of articles that covered a period of more than a decade.

## Conclusion

This scoping review has identified some health system strategies and responses to the effects of CC within the SSA region. The review has revealed that existing strategies and responses are fragmented and hindered by some implementation challenges, such as insufficient funding, lack of human resource capacity, and inadequate surveillance systems. The findings further provide a wider opportunity for policy consideration and formulation of guidelines that can strengthen health systems to respond to CC extreme weather events effectively. As CC continues to pose health threats to global health, urgent and effective interventions are required to minimize its impacts. It is essential to understand the unique vulnerabilities of the health systems, particularly those in the SSA region. The identified strategies and responses are essential for ensuring that health systems continue to provide healthcare services in times of extreme weather events. This calls for more comprehensive, integrated, and well-funded strategies across all six WHO building blocks for health systems (crucial for policymakers and researchers) to strengthen CC response within the SSA region. The time is now to develop strategies and responses that can improve and strengthen health systems as they protect population health against CC risks globally.

## Supporting information

Supplementary Table 1 PRISMA ScR checklist. (DOCX)

Supplementary Table 2 Strings of search words. (DOCX)

Supplementary Table 3 Characteristics of included studies. (DOCX)
