# Supplementary material for: Health system strategies and responses to the effects of Climate Change in Sub-Saharan Africa: A scoping review
Source: PLoS One. 2026 Jun 17;21(6):e0349448. doi: 10.1371/journal.pone.0349448 (PMC13274859; doi:10.1371/journal.pone.0349448)
Supplement: Supplementary Table 2 — (DOCX) [file pone.0349448.s002.docx]

**Supplementary Table 2: Strings of search words in electronic data bases**

| **#** | **Search strings for PubMed**  **Dates of search: From 01/01/2011 - 31/01/25**  **Date of search: 31/01/2025**  **Time of search: 08:00Hrs** | **Results** |
| --- | --- | --- |
| 1 | "health systems" OR "healthcare systems" OR "district health systems" OR "health service systems" | 2,006 |
| 2 | 1+AND "policy strategies" OR "policy responses" OR "policy adaptation" OR "policy mitigation" OR "policy resilience" | 94 |
| 3 | 1+2+AND "Climate Change" OR "extreme weather events" OR "climate variability" OR "Global warming" | 85 |
| 4 | 1+2+3+AND (Africa OR Southern Africa OR East Africa OR Central Africa OR West Africa OR Sub-Saharan Africa OR Sub-Sahara Africa OR Sub Sahara Africa OR AND "Angola" OR "Benin" OR "Botswana" OR "Burkina Faso" OR "Burundi" OR "Cape Verde" OR "Cameroon" OR "Central African Republic" OR "Chad" OR "Comoros" OR "Democratic Republic of the Congo " OR "Republic of the Congo" OR "Cote d'Ivoire" OR "Djibouti" OR "Equatorial Guinea" OR "Eritrea" OR "Ethiopia" OR "Gabon" OR "Gambia" OR "Ghana" OR "Guinea" OR "Guinea-Bissau" OR "Kenya" OR "Lesotho" OR "Liberia" OR "Madagascar" OR "Malawi" OR "Malawi" OR "Mali" OR "Mauritania" OR "Mauritius" OR "Mozambique" OR "Namibia" OR "Niger" OR "Nigeria" OR "Rwanda" OR "Sao Tome and Principe" OR "Senegal" OR "Seychelles" OR "Sierra Leone" OR "Somalia" OR "South Africa" OR "South Sudan" OR "Sudan" OR "Swaziland" OR "Tanzania" OR "Togo" OR "Uganda" OR "Zambia" OR "Zimbabwe” | 9,430 |
|  | **MEDLINE EbscoHost**  **Targeted dates: From 01/01/2011- 31/01/25**  **Date of search: 31/01/2025**  **Time of search: 10:30 Hrs** |  |
| 1 | "health systems" OR "healthcare systems" OR "district health systems" OR "health service systems" | 73,123 |
| 2 | 1+ "policy strategies" OR "policy responses" OR "policy adaptation" OR "policy mitigation" OR "policy resilience" | 158 |
| 3 | 1+2+ "Climate Change" OR "extreme weather events" OR "climate variability" OR "Global warming" | 6 |
| 4 | 1+2+3+"Angola" OR "Benin" OR "Botswana" OR "Burkina Faso" OR "Burundi" OR "Cape Verde" OR "Cameroon" OR "Central African Republic" OR "Chad" OR "Comoros" OR "Democratic Republic of the Congo " OR "Republic of the Congo" OR "Cote d'Ivoire" OR "Djibouti" OR "Equatorial Guinea" OR "Eritrea" OR "Ethiopia" OR "Gabon" OR "Gambia" OR "Ghana" OR "Guinea" OR "Guinea-Bissau" OR "Kenya" OR "Lesotho" OR "Liberia" OR "Madagascar" OR "Malawi" OR "Malawi" OR "Mali" OR "Mauritania" OR "Mauritius" OR "Mozambique" OR "Namibia" OR "Niger" OR "Nigeria" OR "Rwanda" OR "Sao Tome and Principe" OR "Senegal" OR "Seychelles" OR "Sierra Leone" OR "Somalia" OR "South Africa" OR "South Sudan" OR "Sudan" OR "Swaziland" OR "Tanzania" OR "Togo" OR "Uganda" OR "Zambia" OR "Zimbabwe” | 1 |
|  | **Search strings for Scopus:**  **Target dates: From 01/01/2011- 31/01/25**  **Date of search: 31/01/25**  **Time of search: 14:00Hrs** |  |
| 1 | "health systems" OR "healthcare systems" OR "district health systems" OR "health service systems" | 178,667 |
| 2 | 1 + "policy strategies" OR "policy responses" OR "policy adaptation" OR "policy mitigation" OR "policy resilience" | 467 |
| 3 | 1+2+ "Climate Change" OR "extreme weather events" OR "climate variability" OR "Global warming" | 19 |
| 4 | 1+2+3+"Angola" OR "Benin" OR "Botswana" OR "Burkina Faso" OR "Burundi" OR "Cape Verde" OR "Cameroon" OR "Central African Republic" OR "Chad" OR "Comoros" OR "Democratic Republic of the Congo " OR "Republic of the Congo" OR "Cote d'Ivoire" OR "Djibouti" OR "Equatorial Guinea" OR "Eritrea" OR "Ethiopia" OR "Gabon" OR "Gambia" OR "Ghana" OR "Guinea" OR "Guinea-Bissau" OR "Kenya" OR "Lesotho" OR "Liberia" OR "Madagascar" OR "Malawi" OR "Malawi" OR "Mali" OR "Mauritania" OR "Mauritius" OR "Mozambique" OR "Namibia" OR "Niger" OR "Nigeria" OR "Rwanda" OR "Sao Tome and Principe" OR "Senegal" OR "Seychelles" OR "Sierra Leone" OR "Somalia" OR "South Africa" OR "South Sudan" OR "Sudan" OR "Swaziland" OR "Tanzania" OR "Togo" OR "Uganda" OR "Zambia" OR "Zimbabwe” | 1 |
|  | **Search strings for CINAHL**  **Target dates: From 01/01/2011- 31/01/25**  **Date of search: 31/01/25**  **Time of search: 16:00Hrs** |  |
| 1 | ( "health systems" OR "healthcare systems" OR "district health systems" OR "health service systems") | 19,082 |
| 2 | 1+ ("policy strategies" OR "policy responses" OR "policy adaptation" OR "policy mitigation" OR "policy resilience" ) | 58 |
| 3 | 1+2+("Climate Change" OR "extreme weather events" OR "climate variability" OR "Global warming" ) | 2 |
| 4 | 1+2+3+("Angola" OR "Benin" OR "Botswana" OR "Burkina Faso" OR "Burundi" OR "Cape Verde" OR "Cameroon" OR "Central African Republic" OR "Chad" OR "Comoros" OR "Democratic Republic of the Congo " OR "Republic of the Congo" OR "Cote d'Ivoire" OR "Djibouti" OR "Equatorial Guinea" OR "Eritrea" OR "Ethiopia" OR "Gabon" OR "Gambia" OR "Ghana" OR "Guinea" OR "Guinea-Bissau" OR "Kenya" OR "Lesotho" OR "Liberia" OR "Madagascar" OR "Malawi" OR "Malawi" OR "Mali" OR "Mauritania" OR "Mauritius" OR "Mozambique" OR "Namibia" OR "Niger" OR "Nigeria" OR "Rwanda" OR "Sao Tome and Principe" OR "Senegal" OR "Seychelles" OR "Sierra Leone" OR "Somalia" OR "South Africa" OR "South Sudan" OR "Sudan" OR "Swaziland" OR "Tanzania" OR "Togo" OR "Uganda" OR "Zambia" OR "Zimbabwe”) | 0 |
