# Supplementary material for: Health system strategies and responses to the effects of Climate Change in Sub-Saharan Africa: A scoping review
Source: PLoS One. 2026 Jun 17;21(6):e0349448. doi: 10.1371/journal.pone.0349448 (PMC13274859; doi:10.1371/journal.pone.0349448)
Supplement: Supplementary Table 3 — (DOCX) [file pone.0349448.s003.docx]

**Table 3. Characteristics of included articles (n=8).**

| **S/No** | **Author(s), year, location & citation** | **Study title** | **Study design** | **Strategies and responses** | **Implementation Challenges** |
| --- | --- | --- | --- | --- | --- |
| 1 | Nhamo G & Muchuru S, 2019, multi-country [39] | Climate adaptation in the public health sector in Africa: Evidence from United Nations Framework Convention on Climate Change National Communications | Review study | - Adaptable human resource structures and service delivery models. - Strengthening governance and leadership structures - Development of national adaptation plans | - Lack of priority for climate change activities among health workers. - Increased workload during climate change events. |
| 2 | Awuni et al, 2023, Ghana [40] | Managing the challenges of climate change mitigation and adaptation strategies in Ghana | Review study | - Development of a national health adaptation strategy to help address the risk of climate change - Regular data collection for decision making. - Health promotion around communities. - Use solar power energy - Development of resilient infrastructures | - Lack of national assessment of climate change impacts, vulnerability, and adaptation for the health sector. |
| 3 | Koka el al, 2018, Tanzania [41] | Disaster preparedness and response capacity of regional hospitals in Tanzania: a descriptive cross-sectional study | Quantitative study | - Capacity building. - Infrastructure and equipment upgrading and use of an alternative source of power supply. - Formulation of the response committees. - Use of outreach clinics | - Inadequate human resources. - Lack of disaster plans |
| 4 | Opoku et al, 2021, Multi-country [42] | Climate Change and Health Preparedness in Africa: Analyzing Trends in Six African Countries | Quantitative study | - Strengthening of Water and sanitation hygiene activities. Mosquito and vector spraying. Awareness creation using radio and television - Use of early warning systems. - Recruiting surge health workers | - Inadequate funding. - Inadequate knowledge among health workers |
| 5 | Diallo and Ridde, 2024, Senegal [43] | Climate change and resilience of the Senegalese health system in the face of the floods in Keur Massar. | Qualitative study | - The Implementation of interdepartmental synergy. - Develop contingency plan - Redirect resources planned for other activities - Increasing the capacity of health facilities by recruiting additional health workers. - Developing local care outside health establishments through mobile clinics. - Strengthening community ties around collective action. | - Lack of exhaustive mapping of areas with a high climate vulnerability index |
| 6 | Rawat et al, 2022, Ethiopia [44] | The contribution of community health systems to resilience: Case study of the response to the drought in Ethiopia. | Qualitative study | - Involvement of community groups linked to the health system via the primary health care unit. - Building the capacity of the health workforce. - Task shifting - Pharmaceutical infrastructure development - lobby for additional funds from implementing partners and international donors | - Inadequate human resources. - Lack of surveillance and reporting structures. |
| 7 | Chersich MF & Wright CY, 2019, South Africa [45] | Climate change adaptation in South Africa: a case study on the role of the health sector | Systematic review study | - Use of inter-sectoral collaboration. - Capacity building and strengthening community groups. - Research on climate change and health. - Promotion of water, sanitation, and hygiene - Development of early warning systems and risk assessment. | - Inadequate funding, inadequate knowledge among health workers. |
| 8 | Hussey LK & Arku G, 2020, Ghana [46] | Are we ready for it? Health systems preparedness and capacity towards climate change-induced health risks: perspectives of health professionals in Ghana | Mixed-method study | - Training and capacity building. - Setting up early response plans. - Strengthen research capacity. - Use of multi-sectoral collaboration and development of a response plan | - Inadequate funding |
