# Supplementary material for: Health system strategies and responses to the effects of Climate Change in Sub-Saharan Africa: A scoping review
Source: PLoS One. 2026 Jun 17;21(6):e0349448. doi: 10.1371/journal.pone.0349448 (PMC13274859; doi:10.1371/journal.pone.0349448)
Supplement: Attachment — Submitted filename: RESPONSE TO REVIEWERS.docx [file pone.0349448.s005.docx]

**Dear Editor,**

I am very grateful for the insightful review given to our scoping review manuscript entitled “**Health system strategies and responses to the effects of climate change in Sub-Saharan Africa: A scoping review”.** We are very grateful, and we thank all reviewers for their time and effort in reviewing our manuscript. We have considered all comments provided by each of the three reviewers. We sincerely appreciate their comments, as they have improved the manuscript's readability and also scientifically.

Here we provide point-by-point responses to the reviewers’ comments made. Please take note that the line number on this response document corresponds to the manuscript in track changes. We do appreciate the quick review given to our manuscript.

**RESPONSE TO REVIEWERS**

| **REVIEWER1** | | |
| --- | --- | --- |
| **NO** | **COMMENTS** | **RESPONSE** |
| 1 | The paper is technically sound and the data support the conclusions. The authors employed appropriate methods for conducting a scoping review as clearly shown in table 1. However, one of the statements in the conclusion - Prompt efforts should be made to ensure that the effects of CC on health systems are reduced if the region is to achieve the Sustainable Development Goals and the desired African Agenda 2063 - is not derived from the data. | We do appreciate the reviewer for pointing this out. We have removed the sentence “**Prompt efforts should be made to ensure that the effects of CC on health systems are reduced if the region is to achieve the Sustainable Development Goals and the desired African Agenda 2063”**. **See the deleted lines 520-522** |
| 2 | This study did not look at the effects of CC on health systems and the achievement of SDG in Africa, so this extrapolation of conclusions is not valid. | We thank the reviewer for providing this insight, and we have removed the sentence. **See the deleted lines 520-522** |
| 3 | All the data is presented in the manuscript | Thank you very much |
| 4 | This was a simple descriptive qualitative study so did not require statistical analysis. | We thank the reviewer for this important comment. We have only provided background to the number of identified articles and the included articles just to give our readers a snapshot. **See lines 270-274** |
| 5 | The paper is written in clear, easy-to-understand language. | We thank the reviewer for this compliment |
| **REVIEWER 2** | | |
| **ABSTRACT** | | |
| 1 | The abbreviation Sub-Saharan Africa (SSA) should be introduced once and used consistently throughout the abstract and manuscript. Variations such as “Sub-Saharan Region” and “Sub-Saharan African Region” could be avoided. | We thank the reviewer for this insightful suggestion. We have addressed all these suggestions by introducing the abbreviation SSA once. Furthermore, we have addressed the consistency of using “Sub-Saharan African” and “Sub-Saharan Region” as “Sub-Saharan African region”; see lines No. **14-15 and 36-42.** |
| 2 | The abbreviation climate change (CC) is inconsistently used. For example, “Climate Change” appears later without abbreviation. This should be standardized throughout. | Thank you very much for pointing out this error. We have corrected this and an abbreviation has been introduced under introduction while in abstract no abbreviations has been used. **See lines 12, 33 and 34** |
| 3 | The title may not require “The” at the beginning. | Thank you very much. We have corrected this by deleting the word “ The” **see line 1.** |
| 4 | The Methods subsection is incomplete. The abstract does not describe the inclusion and exclusion criteria, an essential component for transparency, especially for a scoping review. | We thank the reviewer for this kind reminder. We have edited the section by adding the following **“We included peer-reviewed articles (original quantitative and qualitative studies, mixed methods studies, “reviews, editorials, and commentaries) published between 2011 and 2025. All book chapters and grey literature (dissertations, conference proceedings, abstracts, and reports) publications primarily focusing on climate change strategies and responses without effects on health systems were excluded” see lines 21-26** |
| 5 | In the Results subsection, the reference to “all building blocks of the health system” needs clarification. If this refers to the six WHO health system building blocks, this should be stated. | We thank the reviewer for this insight. We have included the missing words and now it reads “No study was identified that had outlined strategies and responses across **all six World Health Organisation** building blocks of the health systems**”. See line 31** |
|  | **INTRODUCTION** |  |
| 6 | The introduction requires substantial language editing for clarity, coherence, and flow. Several sentences are long, repetitive, or conceptually unclear. | We thank the reviewer, and we have edited the whole part of introduction |
| 7 | There is confusion about what the manuscript identifies as the primary stressor in SSA, climate change, poverty, or overlapping systemic vulnerabilities. The narrative shifts without clear positioning. | We thank the reviewer for identifying this. The primary stressor the manuscript is addressing is climate change, while the other included are extreme events resulting from climate change. This being the case, we have edited all areas throughout the manuscript. |
| 8 | The claim that climate change “exposes humans to pandemics and epidemics” is made without a supporting reference. This type of statement needs scientific grounding. | We thank the reviewer for pointing out this. We have added the reference now. **See line 77-78** |
| 9 | Paragraphs that attempt to explain the relationship between climate change and health systems (particularly paragraph 3) are difficult to follow and require restructuring for conceptual and language clarity. | We thank the reviewer for noticing this. We have restructured the paragraph as suggested. |
| 10 | Subsequent paragraphs also lack coherence and do not clearly articulate the problem, the evidence gap, or the rationale for conducting this review. | Thanks for highlighting this. We have restructured all paragraphs. **See lines 79- 97, and 98 to 112** |
| 11 | Major section headers such as introduction, methods, results, discussion, and conclusion should be uppercase to distinguish them from subheadings. | We thank the reviewer for suggesting this. However, we could not do this as we were following the requirements by the journal. |
| 12 | The document contains unnecessary subheadings (e.g., a separate “Protocol Registration Status”), which are more appropriate within the Methods narrative. The manuscript should reflect scientific writing conventions rather than technical report formatting. | We thank the reviewer for pointing out this. We have moved the registration statement to methods. **See lines 131-132** |
|  | **METHODS** |  |
| 13 | The database selection is limited. A scoping review on SSA health system responses should include a broader range of databases relevant to the region (e.g., EMBASE, Web of Science, Africa Index Medicus, regional and policy databases). | We thank the reviewer for recommending this point. At initial stage, we consulted widely, including librarians and some researchers with good track records on scoping review. During the consultations we were advised that for the scoping review to be trust worth, atleast four databases are enough. However, taking into consideration of this suggestion, we have searched and included another database of African Index Medicus and two more repositories, ATACH Community Country Documents Repository and ClimHealth Africa Resources. **See the PRISMA** |
| 14 | The grey literature search is insufficient. Limiting grey literature to WHO websites does not capture national policies, NGO reports, or locally generated evidence that are central to understanding SSA health system strategies. | We thank the reviewer for pointing out this. We have added to our search for grey literature the Clim-Health Africa Resources and Alliance for Transformative Action on Climate and Health Repositories. **See the PRISMA** |
| 15 | The manuscript does not provide full, reproducible search strings. | We thank the reviewer for this. We were mindful about word limit, and as such, we provided full search strings in supplementary table 2. However, to address this concern, we have added search strings as appendix 1 immediately after references. **See line 806-808** |
| 16 | Search dates are incompletely reported. It only says “the search was completed on January 31, 2025”. | We thank the reviewer for highlighting this. We have edited and included the date when the search began. Now it reads “The search was conducted multiple times from November 1, 2024, and was completed on January 31, 2025”. **See lines 149-150** |
| 17 | Overlap between PubMed and MEDLINE is not acknowledged or justified. PubMed search yielded 9430 studies whilst MEDLINE yielded only 1 study. | We thank the reviewer for pointing out this. It is true that PubMed and Medline are one, so we have combined the identified articles. **See the PRISMA diagram lines 213-216** |
| 18 | The decision to include commentaries, editorials, and reviews without clear justification weakens the evidence base. | We thank the reviewer for identifying this. We have provided the following justification “**to capture and map a comprehensive overview of the available information and opinions on the strategies and responses”. See lines 152-155** |
| 19 | Excluding non-English articles due to “lack of funds” introduces significant bias and needs proper acknowledgment or alternative mitigation. | We thank the reviewer for this. We have removed language as a limitation for the study. This has been addressed based on the recommendation to use an Artificial Intelligence (AI) word translator. We went back into the Covidence software, where we retrieved all rejected non-English language articles. In total, we retrieved 13 articles, which we translated into English, assisted by AI as per the reviewer’s advice. However, none of the retrieved articles had strategies and responses to climate change effects on the health system**. See lines 152-155** |
| 20 | The process for resolving screening disagreements is missing. | We thank the reviewer for noticing this. We have indicated how screening disagreements were resolved by including the following sentence; “**All screening disagreements between the two reviewers, CSC and PH, were resolved by inviting the third reviewer (SM) who resolved**”. **See lines 169-170** |
| 21 | The data extraction process is inadequately described. There is no mention of piloting extraction tools. | We thank the reviewer for noticing this. We have included pilot testing statement under data extraction which reads, **“Pilot testing was performed before the extraction to ensure inter-rater agreement among the reviewers**”. **See lines 245-246** |
| 22 | The rationale for mapping findings to the WHO building blocks is not explained, and alternative frameworks are not considered. | We thank the reviewer for this point. We have added clarification or rationale for mapping the findings with the WHO building blocks as follows: **“Findings are presented in themes aligned with the six WHO building blocks of the health systems for an in-depth understanding on how each building block is being strengthened in response to CC events”** under results, sub-selected studies. **See lines 279-281** |
|  | **RESULTS** |  |
| 23 | There are inconsistencies in the PRISMA numbers, textual descriptions, and tables. These discrepancies undermine confidence in the accuracy of the review process. | We thank the reviewer for identifying this. We have corrected this error. **See lines 283-286, PRISMA and Table 2** |
| 24 | Table numbers do not match their in-text citations. | We thank the reviewer for this comment. Indeed, there were mismatch on the citation which we have corrected in the table and the text. **See table 2 and lines 283-286 and 304-374** |
| 25 | Some included studies appear to be from countries outside SSA (e.g., Egypt) with no explanation. | We thank the reviewer for this observation. We have added justification on this which reads; **“This study was included as it presented study findings from each country separately and we did not include findings from Egypt”**. **See lines 290-292** |
| 26 | The results section largely lists strategies rather than synthesizing or analyzing them. | We thank the reviewer for noting this. We do appreciate that the main aim for this scoping review was to find the strategies and responses with little focus on synthesizing them. We could have gone that far if it was a systematic review, however, we have learnt something out this comment and we thank the reviewer for this. |
| 27 | The mapping of findings to the WHO building blocks feels forced in some cases, and some listed strategies do not reflect true “health system strategies.” | We thank the reviewer on this. We used inductive approach of which we did not have pre-identified strategy and response themes. The responses and strategies emerged from the articles, of which some articles specified on the WHO building block while others did not. We sought an assistance from health system experts who assisted in assigning the strategies and responses identified from the articles to six WHO building blocks for the health system. **See lines 299-302** |
| 28 | The "characteristics of included articles" table lacks essential details. | We thank the reviewer. We have added the missing essential details including author(s), year, location, citation, study title, study design, Strategies and responses and Implementation Challenges**. See table 2** |
|  | **DISCUSSION** |  |
| 29 | The discussion section tends to repeat results rather than interpreting them | Thank for this feedback. We have edited the whole chapter based on this comment. |
| 30 | The conceptual framing is unfocused. The narrative jumps between climate adaptation, health system resilience, environmental disasters, and emergency preparedness without a clear thread. | We thank the reviewer for this. We have entirely worked out this concern throughout the manuscript to align with the main focus on strategies and responses |
| 31 | There is no assessment of the quality, strength, or limitations of the included evidence. | We thank the reviewer for the insight. The included papers were appraised by two independent reviewers, and then address the discrepancies were addressed. All the articles included were appraised highly. We have included this under appraisal of selected studies. **See lines 275-279** |
| 32 | Key limitations, including the narrow search strategy, English-only restriction, and limited grey literature, are not sufficiently acknowledged. | We thank the reviewer for pointing out this. We have addressed all these. On search strategies, we have added one more. On the restriction to English articles only, this has been addressed, as we retrieved all articles that we rejected because of other languages and managed to translate them using AI. On grey literature, we have searched more repositories; no article met our inclusion criteria. **See the deleted lines 495-500 also 155-157 under eligibility criteria** |
| 33 | Policy implications remain broad and disconnected from the actual evidence extracted. | We thank the reviewer for this point. We have narrowed to strategies and responses as main focus for policy implication |
|  | **CONCLUSION** |  |
| 34 | The conclusion partially repeats earlier sections and introduces new general statements rather than summarizing grounded findings. The conclusion should be more concise and aligned with what the study actually demonstrates. | We thank the reviewer for this. We have restructured the conclusion as suggested. **See lines 507-526** |
|  | **PRESENTATION AND LANGUAGE QUALITY** |  |
| 35 | The manuscript requires significant editing for grammar, clarity, structure, and readability, particularly the results and discussion sections. | We thank the reviewer and this has been done throughout the manuscript. |
| 36 | Line numbers are missing from the submitted manuscript, which complicates the review process. | Thanks for this and we have provided line numbers in the track change document. |
| **REVIEWER 3** | | |
| **FORM AND PRESENTATION** | | |
| 1 | The use of et al. is inconsistent across citations with more than six authors. According to the Uniform Requirements for Manuscripts, this should be standardized. | We thank our reviewer for noting this. We edited all citations, and those with more than six authors have been standardized accordingly. **See lines 551-804** |
| 2 | Reference 56 is missing its publication year. | We thank the reviewer for noticing this, and we have removed the reference from our list. **See line 775-776** |
| 3 | Several in-text citations (e.g., references 39, 40, and 41 in Table 2) do not correspond to the numbering in the reference list. This inconsistency may also affect other citations throughout the manuscript and should be carefully corrected. | Thank you very much for noticing this. We have corrected the numbers and the table. **See table 2 and lines 283-286 and 304-374** |
|  | **CONTENT AND SOURCES** |  |
| 1 | The paper addresses the question: “What are the health system strategies and responses to the effects of climate change in Sub-Saharan Africa?”  While health system responses, understood as concrete adaptation or resilience-building activities, can be documented through scientific publications, identifying health system strategies, defined as strategic and planning documents, is more challenging. Relying solely on peer-reviewed papers is not the most appropriate approach.  Grey literature, particularly WHO country support documents, national health system plans, and climate-health reports, provides more reliable evidence for strategic planning.  Databases such as PubMed, CINAHL, Scopus, and MEDLINE are not suitable sources for grey literature. Instead, repositories such as:  1. ATACH Community Country Documents Repository (<https://www.atachcommunity.com/resources/country-documents-repository/>) 2. ClimHealth Africa Resources (<https://climhealthafrica.org/resources/>)  should be consulted to capture relevant planning documents and reports. | We thank the reviewer for this suggestion. We searched the repositories provided in addition to WHO; however, no article meeting the study inclusion criteria was obtained. **See the PRISMA** |
| 2 | References 1 to 4 can be updated by recent releases from Lancet Countdown, IPCC AR6 and many other papers focusing on health impact of CC | We thank the reviewer for this suggestion. We have updated the 4 references. **See lines 551-578** |
|  | **LANGUAGE CONSIDERATION** |  |
| 1 | The manuscript focuses only on English-language sources.  In the current era of AI-assisted translation, language barriers are no longer a strong justification for restricting the review to English. Including documents at least in French and Portuguese would strengthen the comprehensiveness of the study. | We thank the reviewer for pointing this out. We went back into the Covidence software, where we retrieved all rejected non-English language articles. In total, we retrieved 13 articles, which we translated into English, assisted by AI as per the reviewer’s advice. However, none of the retrieved articles had strategies and responses to climate change effects on the health system**. See the PRISMA** |
|  | **SUMMARY OF KEY REVISIONS** |  |
| 1 | Update and standardize reference formatting and correct inconsistencies | We thank the reviewer for this advice. We have updated and standardized the references. **See lines 551-804** |
| 2 | Incorporate grey literature sources for health system strategies and planning. | We thank you for this advice. We have considered this. See the PRISMA |
| 3 | Expand beyond English-only sources by leveraging translation tools. | Thanks a lot for this. This suggestion was an eye-opener. We went back into the Covidence software, where we retrieved all article rejected because of languages. Using Artificial Intelligent language translator, we translated them. Unfortunately, none meet our eligibility criteria. |
