# Supplementary material for: Health system strategies and responses to the effects of Climate Change in Sub-Saharan Africa: A scoping review
Source: PLoS One. 2026 Jun 17;21(6):e0349448. doi: 10.1371/journal.pone.0349448 (PMC13274859; doi:10.1371/journal.pone.0349448)
Supplement: Attachment — Submitted filename: RESPONSE TO EDITOR.docx [file pone.0349448.s006.docx]

**Dear Editor,**

Receive our greetings.

I am very grateful for the insightful review given to our scoping review manuscript entitled “**Health system strategies and responses to the effects of climate change in Sub-Saharan Africa: A scoping review”.** We are very grateful, and we thank you for the time and the effort taken in reviewing our manuscript. We have considered all comments and suggestions provided carefully to improve the manuscript's readability and scientific presentation.

Here we provide point-by-point responses to the comments and suggestions made. Please take note that the line numbers on this response document corresponds to the manuscript in track changes while page numbers on the PRISMA ScR check list correspond to the cleaned document. We do appreciate the quick review given to our manuscript.

**RESPONSE TO EDITOR**

| **INTRODUCTION** | | |
| --- | --- | --- |
| **NO** | **COMMENTS** | **RESPONSE** |
| 1 | Paragraph 1: Define the health system, explaining its dimensions at both facility and community levels; describe its building blocks; introduce the concept of health system resilience in relation to climate change; and outline factors that support resilience. | We do appreciate for suggesting this. We have restructured this paragraph by;   1. Defining the health system 2. Proving dimensions from community to facility level 3. Describing the six building blocks 4. Introducing the climate change concept and 5. Highlighting the concept of resilience and factors that support the concept. **See lines 45-64** |
| 2 | Paragraph 2: Define climate change and describe its potential impact and implications for health systems and their resilience. | We thank you for suggesting this, and we have restructured the paragraph by   1. Define climate change 2. Describe its potential impacts 3. Indicating its implications for the health systems and their resilience. **See lines 65-85** |
| 3 | Paragraph 3: Review existing evidence on health system resilience to climate change, highlighting what is known and the evidence gaps this scoping review intends to address. | We thank you for this constructive suggestion. We have provided evidence on health system resilience to climate change by highlighting what is already known and the gap our study intends to address. **See lines 87-104** |
| 4 | Paragraph 4: State the research objectives and guiding questions. | We thank you for pointing this. We have restructured this paragraph as suggested. Please **see lines 105-113** |
| **RESULTS** | | |
| 1 | The current PRISMA ScR diagram requires redrawing for better clarity and completeness, ensuring that all elements raised by Reviewer 2 are fully incorporated.  **Reviewer 2** also noticed the inconsistencies in the PRISMA numbers, textual descriptions, and tables. These discrepancies undermine confidence in the accuracy of the review process. | We thank you for this suggestion. We have revised the PRISMA ScR diagram as suggested, and we have also incorporated suggestions made by reviewer 2. **Please see the PRISMA ScR diagram, Supplementary Table 1 and the referred pages.**  WE have addressed all inconsistencies in the PRISMA numbers, textual descriptions, and tables. **See PRISMA Supplementary Figure 1, supplementary Table 4 and lines 240-249** |
| 2 | Table 1 appears to be missing; you may consider relabeling the current Table 2 accordingly. | Thank you very much for noticing this error. We have corrected this and relabeled the table accordingly. **See supplementary Table 4** |
| 3 | Content in lines 275–279 should be moved to the Methods section, as the Results should focus exclusively on reporting outcomes of the quality assessment and findings. | Thank you very much for this advice. We have moved this statement to the methods section as suggested. **Please see lines 227-231.** |
|  | **DISCUSSION** |  |
| 1 | Paragraph 1: Summarize the study objectives and key findings, including the breadth of available literature, evidence related to the health system building blocks (governance and leadership, human resources, health information management, and service delivery), the limited evidence on health financing and essential medicines/health technologies, and the challenges identified. | We thank you very much for suggesting this. We have restructured the paragraph as suggested. **Please see lines 338-353** |
| 2 | Paragraphs 2–4: Provide a detailed discussion of findings, exploring the factors that may explain observed trends and comparing them with results from other studies. | We thank you for this suggestion and advice. We have restructured paragraphs 2-4. **Please see lines 354-445** |
| 3 | Paragraph 5: Present the identified evidence gaps and discuss their implications for future research. | We thank you for this suggestion. We have restructured this paragraph accordingly. **Please see lines 446-490** |
| 4 | Paragraph 6: Outline the study’s limitations. | We thank you for this. We have restructured the paragraph accordingly. **Please see lines 493-501.** |

Kind regards,

**Chancy Chimatiro**

**Corresponding Author**
