# Supplementary material for: Health system strategies and responses to the effects of Climate Change in Sub-Saharan Africa: A scoping review
Source: PLoS One. 2026 Jun 17;21(6):e0349448. doi: 10.1371/journal.pone.0349448 (PMC13274859; doi:10.1371/journal.pone.0349448)
Supplement: Attachment — Submitted filename: RESPONSE TO EDITOR-2.docx [file pone.0349448.s007.docx]

**Dear Editor,**

Receive our greetings.

We greatly appreciate the quick feedback and review given to our manuscript entitled “**Health system strategies and responses to the effects of climate change in Sub-Saharan Africa: A scoping review”.** We thank you for the time and the effort taken. We have considered all current comments made and suggestions provided carefully to further improve our manuscript.

Here we provide point-by-point responses to the comments and suggestions made.

**RESPONSE TO EDITOR**

| **INTRODUCTION** | | |
| --- | --- | --- |
| **NO** | **COMMENTS** | **RESPONSE** |
| 1 | Why are all tables and figures included as supplementary? At the least, the current supplementary figure 1 and supplementary table 3 should be included in the manuscript text. | Thanks for asking this question.  To rectify this, by indicating Figure 1 in the manuscript. **See page 6 line 147 cleaned manuscript and page 6 line 148 for manuscript with track changes**.  And Table 4 in the manuscript. **See pages 7 for the cleaned manuscript line 167 and 186 and 7-8 of the manuscript with track changes lines 168 and 192** |
| 2 | No mention is made of supplementary tables 1-2 in the manuscript text? Where are they? | We kindly thank you for this. Supplementary table 1 is mentioned under “materials and methods” page 5. **See line 107, page 5 of both cleaned manuscript and manuscript with track changes.**  Supplementary table 2 is mentioned under “Identification of relevant studies and search strategy”. **See line 127, page 6 for cleaned and track changed manuscript** |
| 3 | Lines 244-249 fits more in the methods section. You should then present the result of the MMAT in the result section. | We thank you for this. We have moved the line to study selection. **See lines 133-137, page 6 of both clean and track change manuscripts** |
| 4 | In line 245, please correct the referencing style to be consistent with the rest of the document. | Thanks for pointing this out. We have corrected the referencing style as advised. |
| 5 | Please introduce a sub-heading in line 270 (just before service delivery) to read "Health system building blocks strategies for mitigating the impact of climate change" | Thanks for this insight advise. We have introduced the subheading “Health system building blocks strategies for mitigating the impact of climate change”. **See line 198 of cleaned manuscript and 207 of the manuscript with track changes** |
| 6 | Line 436-443 is unclear. Is this conclusion from the finding of your review or the finding of other studies? Please make this clearer | Thanks for pointing out this. You are right, this conclusion was from the findings of other studies that reported similar findings to our findings. We have edited and now it reads **“Elsewhere, the use of health information technologies has been reported to strengthen health systems’ ability to predict and project the occurrence of CC-related extreme events which prompt preparedness for effective response [50]. In addition, another study reported that health information technologies, such as early warning systems, provide opportunities to detect potential health risks and aid early response preparedness before health hazards occur [50]. It further reported that if used properly, early warning systems may reduce financial costs and protect vulnerable health systems from catastrophic financial spending [50]. Furthermore, similar findings were also reported in another study by Rahimi-Ardabili et al (2022), in their review on digital health for climate change mitigation and response [51]”. See lines 290-298 in cleaned manuscript and lines 300-309 in the manuscript with track changes.** |
| 7 | Further copy-editing and proof reading of the manuscript is required to correct some errors that may have been included in the use of track change mode. | We thank you for this. We have done this as per your advice. |

Kind regards,

**Chancy Chimatiro**

**Corresponding Author**
