# Supplementary material for: Health system strategies and responses to the effects of Climate Change in Sub-Saharan Africa: A scoping review
Source: PLoS One. 2026 Jun 17;21(6):e0349448. doi: 10.1371/journal.pone.0349448 (PMC13274859; doi:10.1371/journal.pone.0349448)
Supplement: Peer review history

# Peer review: Decision Letter 0

**DOI:** 10.1371/journal.pone.0349448.r001

**Authors:** Olushayo Olu

13 Jan 2026

PONE-D-25-44483

The health systems strategies and responses to the effects of Climate Change in Sub-Saharan Africa: A scoping review

PLOS One

Dear Dr. Chimatiro,

Thank you for submitting your manuscript to PLOS ONE. After careful consideration, we feel that it has merit but does not fully meet PLOS ONE’s publication criteria as it currently stands. Therefore, we invite you to submit a revised version of the manuscript that addresses the points raised during the review process.

Please submit your revised manuscript by Feb 27 2026 11:59PM. If you will need more time than this to complete your revisions, please reply to this message or contact the journal office at plosone@plos.org. When you're ready to submit your revision, log on to https://www.editorialmanager.com/pone/ and select the 'Submissions Needing Revision' folder to locate your manuscript file.

Please include the following items when submitting your revised manuscript:

- A letter that responds to each point raised by the academic editor and reviewer(s). You should upload this letter as a separate file labeled 'Response to Reviewers'.
- A marked-up copy of your manuscript that highlights changes made to the original version. You should upload this as a separate file labeled 'Revised Manuscript with Track Changes'.
- An unmarked version of your revised paper without tracked changes. You should upload this as a separate file labeled 'Manuscript'.

If you would like to make changes to your financial disclosure, please include your updated statement in your cover letter. Guidelines for resubmitting your figure files are available below the reviewer comments at the end of this letter.

If applicable, we recommend that you deposit your laboratory protocols in protocols.io to enhance the reproducibility of your results. Protocols.io assigns your protocol its own identifier (DOI) so that it can be cited independently in the future. For instructions see: https://journals.plos.org/plosone/s/submission-guidelines#loc-laboratory-protocols. Additionally, PLOS ONE offers an option for publishing peer-reviewed Lab Protocol articles, which describe protocols hosted on protocols.io. Read more information on sharing protocols at https://plos.org/protocols?utm_medium=editorial-email&utm_source=authorletters&utm_campaign=protocols.

We look forward to receiving your revised manuscript.

Kind regards,

Olushayo Oluseun Olu

Academic Editor

PLOS One

Journal requirements:

When submitting your revision, we need you to address these additional requirements.

1. Please ensure that your manuscript meets PLOS ONE's style requirements, including those for file naming. The PLOS ONE style templates can be found at

https://journals.plos.org/plosone/s/file?id=wjVg/PLOSOne_formatting_sample_main_body.pdf and

https://journals.plos.org/plosone/s/file?id=ba62/PLOSOne_formatting_sample_title_authors_affiliations.pdf

2. When completing the data availability statement of the submission form, you indicated that you will make your data available on acceptance. We strongly recommend all authors decide on a data sharing plan before acceptance, as the process can be lengthy and hold up publication timelines. Please note that, though access restrictions are acceptable now, your entire data will need to be made freely accessible if your manuscript is accepted for publication. This policy applies to all data except where public deposition would breach compliance with the protocol approved by your research ethics board. If you are unable to adhere to our open data policy, please kindly revise your statement to explain your reasoning and we will seek the editor's input on an exemption. Please be assured that, once you have provided your new statement, the assessment of your exemption will not hold up the peer review process.

3. Please include captions for your Supporting Information files at the end of your manuscript, and update any in-text citations to match accordingly. Please see our Supporting Information guidelines for more information: http://journals.plos.org/plosone/s/supporting-information.

If the reviewer comments include a recommendation to cite specific previously published works, please review and evaluate these publications to determine whether they are relevant and should be cited. There is no requirement to cite these works unless the editor has indicated otherwise.

[Note: HTML markup is below. Please do not edit.]

Reviewers' comments:

Reviewer's Responses to Questions

**Comments to the Author**

1. Is the manuscript technically sound, and do the data support the conclusions?

The manuscript must describe a technically sound piece of scientific research with data that supports the conclusions. Experiments must have been conducted rigorously, with appropriate controls, replication, and sample sizes. The conclusions must be drawn appropriately based on the data presented.

Reviewer #1: Yes

Reviewer #2: No

Reviewer #3: Partly

**********

2. Has the statistical analysis been performed appropriately and rigorously?

Reviewer #1: N/A

Reviewer #2: I Don't Know

Reviewer #3: N/A

**********

3. Have the authors made all data underlying the findings in their manuscript fully available?

The PLOS Data policy requires authors to make all data underlying the findings described in their manuscript fully available without restriction, with rare exception (please refer to the Data Availability Statement in the manuscript PDF file). The data should be provided as part of the manuscript or its supporting information, or deposited to a public repository. For example, in addition to summary statistics, the data points behind means, medians and variance measures should be available. If there are restrictions on publicly sharing data—e.g. participant privacy or use of data from a third party—those must be specified.

Reviewer #1: Yes

Reviewer #2: No

Reviewer #3: Yes

**********

4. Is the manuscript presented in an intelligible fashion and written in standard English?

PLOS ONE does not copyedit accepted manuscripts, so the language in submitted articles must be clear, correct, and unambiguous. Any typographical or grammatical errors should be corrected at revision, so please note any specific errors here.

Reviewer #1: Yes

Reviewer #2: No

Reviewer #3: Yes

**********

5. Review Comments to the Author

Please use the space provided to explain your answers to the questions above. You may also include additional comments for the author, including concerns about dual publication, research ethics, or publication ethics. (Please upload your review as an attachment if it exceeds 20,000 characters)

Reviewer #1: The paper is technically sound and the data support the conclusions. The authors employed appropriate methods for conducting a scoping review as clearly shown in table 1. However one of the statements in the conclusion - Prompt efforts should be made to ensure that the effects of CC on health systems are reduced if the region is to achieve the Sustainable Development Goals and the desired African Agenda 2063 - is not derived from the data. This study did not look at the effects of CC on health systems and the achievement of SDG in Africa, so this extrapolation of conclusions is not valid. All the data is presented in the manuscript. This was a simple descriptive qualitative study so did not require statistical analysis. The paper is written in clear, easy-to-understand language.

Reviewer #2: The topic, “The health system strategies and responses to climate change in Sub-Saharan Africa: a scoping review” is important and timely. However, the manuscript in its current form has substantial conceptual, methodological, structural, and language-related problems that limit its scientific contribution.

ABSTRACT

1. The abbreviation Sub-Saharan Africa (SSA) should be introduced once and used consistently throughout the abstract and manuscript. Variations such as “Sub-Saharan Region” and “Sub-Saharan African Region” could be avoided.

2. The abbreviation climate change (CC) is inconsistently used. For example, “Climate Change” appears later without abbreviation. This should be standardized throughout.

3. The title may not require “The” at the beginning.

4. The Methods subsection is incomplete. The abstract does not describe the inclusion and exclusion criteria, an essential component for transparency, especially for a scoping review.

5. In the Results subsection, the reference to “all building blocks of the health system” needs clarification. If this refers to the six WHO health system building blocks, this should be stated.

INTRODUCTION

6. The introduction requires substantial language editing for clarity, coherence, and flow. Several sentences are long, repetitive, or conceptually unclear.

7. There is confusion about what the manuscript identifies as the primary stressor in SSA, climate change, poverty, or overlapping systemic vulnerabilities. The narrative shifts without clear positioning.

8. The claim that climate change “exposes humans to pandemics and epidemics” is made without a supporting reference. This type of statement needs scientific grounding.

9. Paragraphs that attempt to explain the relationship between climate change and health systems (particularly paragraph 3) are difficult to follow and require restructuring for conceptual and language clarity.

10. Subsequent paragraphs also lack coherence and do not clearly articulate the problem, the evidence gap, or the rationale for conducting this review.

11. Major section headers such as introduction, methods, results, discussion, and conclusion should be uppercase to distinguish them from subheadings.

12. The document contains unnecessary subheadings (e.g., a separate “Protocol Registration Status”), which are more appropriate within the Methods narrative. The manuscript should reflect scientific writing conventions rather than technical report formatting.

METHODS

This section has major methodological shortcomings that affect the credibility and reproducibility of the review.

13. The database selection is limited. A scoping review on SSA health system responses should include a broader range of databases relevant to the region (e.g., EMBASE, Web of Science, Africa Index Medicus, regional and policy databases).

14. The grey literature search is insufficient. Limiting grey literature to WHO websites does not capture national policies, NGO reports, or locally generated evidence that are central to understanding SSA health system strategies.

15. The manuscript does not provide full, reproducible search strings.

16. Search dates are incompletely reported. It only says “the search was completed on January 31, 2025”.

17. Overlap between PubMed and MEDLINE is not acknowledged or justified. PubMed search yielded 9430 studies whilst MEDLINE yielded only 1 study.

18. The decision to include commentaries, editorials, and reviews without clear justification weakens the evidence base.

19. Excluding non-English articles due to “lack of funds” introduces significant bias and needs proper acknowledgment or alternative mitigation.

20. The process for resolving screening disagreements is missing.

21. The data extraction process is inadequately described. There is no mention of piloting extraction tools.

22. The rationale for mapping findings to the WHO building blocks is not explained, and alternative frameworks are not considered.

RESULTS

23. There are inconsistencies in the PRISMA numbers, textual descriptions, and tables. These discrepancies undermine confidence in the accuracy of the review process.

24. Table numbers do not match their in-text citations.

25. Some included studies appear to be from countries outside SSA (e.g., Egypt) with no explanation.

26. The results section largely lists strategies rather than synthesizing or analyzing them.

27. The mapping of findings to the WHO building blocks feels forced in some cases, and some listed strategies do not reflect true “health system strategies.”

28. The "characteristics of included articles" table lacks essential details.

DISCUSSION

29. The discussion section tends to repeat results rather than interpreting them.

30. The conceptual framing is unfocused. The narrative jumps between climate adaptation, health system resilience, environmental disasters, and emergency preparedness without a clear thread.

31. There is no assessment of the quality, strength, or limitations of the included evidence.

32. Key limitations, including the narrow search strategy, English-only restriction, and limited grey literature, are not sufficiently acknowledged.

33. Policy implications remain broad and disconnected from the actual evidence extracted.

CONCLUSION

34. The conclusion partially repeats earlier sections and introduces new general statements rather than summarizing grounded findings. The conclusion should be more concise and aligned with what the study actually demonstrates.

PRESENTATION AND LANGUAGE QUALITY

35. The manuscript requires significant editing for grammar, clarity, structure, and readability, particularly the results and discussion sections.

36. Line numbers are missing from the submitted manuscript, which complicates the review process.

Reviewer #3: FORM AND PRESENTATION

The manuscript is generally well structured and adheres to the journal’s formatting requirements. However, there are some weaknesses in the reference section:

1. The use of et al. is inconsistent across citations with more than six authors. According to the Uniform Requirements for Manuscripts, this should be standardized.

2. Reference 56 is missing its publication year.

3. Several in-text citations (e.g., references 39, 40, and 41 in Table 2) do not correspond to the numbering in the reference list. This inconsistency may also affect other citations throughout the manuscript and should be carefully corrected.

CONTENT AND SOURCES

The paper addresses the question: “What are the health system strategies and responses to the effects of climate change in Sub-Saharan Africa?”

While health system responses, understood as concrete adaptation or resilience-building activities, can be documented through scientific publications, identifying health system strategies, defined as strategic and planning documents, is more challenging. Relying solely on peer-reviewed papers is not the most appropriate approach.

Grey literature, particularly WHO country support documents, national health system plans, and climate-health reports, provides more reliable evidence for strategic planning.

Databases such as PubMed, CINAHL, Scopus, and MEDLINE are not suitable sources for grey literature. Instead, repositories such as:

1. ATACH Community Country Documents Repository (https://www.atachcommunity.com/resources/country-documents-repository/)

2. ClimHealth Africa Resources (https://climhealthafrica.org/resources/)

should be consulted to capture relevant planning documents and reports.

References 1 to 4 can be updated by recent releases from Lancet Countdown, IPCC AR6 and many other papers focusing on health impact of CC

LANGUAGE CONSIDERATION

The manuscript focuses only on English-language sources.

In the current era of AI-assisted translation, language barriers are no longer a strong justification for restricting the review to English. Including documents at least in French and Portuguese would strengthen the comprehensiveness of the study.

SUMMARY OF KEY REVISIONS IF the SCOPING REVIEW IS ACCEPTED IN PLOS ONE

1. Update and standardize reference formatting and correct inconsistencies.

2. Incorporate grey literature sources for health system strategies and planning.

3. Expand beyond English-only sources by leveraging translation tools.

**********

6. PLOS authors have the option to publish the peer review history of their article (what does this mean?). If published, this will include your full peer review and any attached files.

If you choose “no”, your identity will remain anonymous but your review may still be made public.

**Do you want your identity to be public for this peer review?**  For information about this choice, including consent withdrawal, please see our Privacy Policy.

Reviewer #1: **Yes:**  Jayne Byakika Tusiime

Reviewer #2: **Yes:**  Robert Lubajo

Reviewer #3: No

**********

[NOTE: If reviewer comments were submitted as an attachment file, they will be attached to this email and accessible via the submission site. Please log into your account, locate the manuscript record, and check for the action link "View Attachments". If this link does not appear, there are no attachment files.]

To ensure your figures meet our technical requirements, please review our figure guidelines: https://journals.plos.org/plosone/s/figures

You may also use PLOS’s free figure tool, NAAS, to help you prepare publication quality figures: https://journals.plos.org/plosone/s/figures#loc-tools-for-figure-preparation.

NAAS will assess whether your figures meet our technical requirements by comparing each figure against our figure specifications.

# Author comment: Author response to Decision Letter 1

**DOI:** 10.1371/journal.pone.0349448.r002

2 Mar 2026

Dear Editor,

I am very grateful for the insightful review given to our scoping review manuscript entitled “Health system strategies and responses to the effects of climate change in Sub-Saharan Africa: A scoping review”. We are very grateful, and we thank all reviewers for their time and effort in reviewing our manuscript. We have considered all comments provided by each of the three reviewers. We sincerely appreciate their comments, as they have improved the manuscript's readability and also scientifically.

Here we provide point-by-point responses to the reviewers’ comments made. Please take note that the line number on this response document corresponds to the manuscript in track changes. We do appreciate the quick review given to our manuscript.

Attachment Submitted filename: RESPONSE TO REVIEWERS.docx

# Editor report: Decision Letter 1

**DOI:** 10.1371/journal.pone.0349448.r003

**Authors:** Olushayo Olu

19 Mar 2026

PONE-D-25-44483R1

Health system strategies and responses to the effects of climate change in Sub-Saharan Africa: A scoping review

PLOS One

Dear Dr. Chimatiro,

Thank you for submitting your manuscript to PLOS ONE. After careful consideration, we feel that it has merit but does not fully meet PLOS ONE’s publication criteria as it currently stands. Therefore, we invite you to submit a revised version of the manuscript that addresses the points raised during the review process.

Please submit your revised manuscript by May 03 2026 11:59PM. If you will need more time than this to complete your revisions, please reply to this message or contact the journal office at plosone@plos.org. When you're ready to submit your revision, log on to https://www.editorialmanager.com/pone/ and select the 'Submissions Needing Revision' folder to locate your manuscript file.

Please include the following items when submitting your revised manuscript:

- A letter that responds to each point raised by the academic editor and reviewer(s). You should upload this letter as a separate file labeled 'Response to Reviewers'.
- A marked-up copy of your manuscript that highlights changes made to the original version. You should upload this as a separate file labeled 'Revised Manuscript with Track Changes'.
- An unmarked version of your revised paper without tracked changes. You should upload this as a separate file labeled 'Manuscript'.

If you would like to make changes to your financial disclosure, please include your updated statement in your cover letter. Guidelines for resubmitting your figure files are available below the reviewer comments at the end of this letter.

If applicable, we recommend that you deposit your laboratory protocols in protocols.io to enhance the reproducibility of your results. Protocols.io assigns your protocol its own identifier (DOI) so that it can be cited independently in the future. For instructions see: https://journals.plos.org/plosone/s/submission-guidelines#loc-laboratory-protocols. Additionally, PLOS ONE offers an option for publishing peer-reviewed Lab Protocol articles, which describe protocols hosted on protocols.io. Read more information on sharing protocols at https://plos.org/protocols?utm_medium=editorial-email&utm_source=authorletters&utm_campaign=protocols.

We look forward to receiving your revised manuscript.

Kind regards,

Olushayo Oluseun Olu

Academic Editor

PLOS One

Journal Requirements:

1. If the reviewer comments include a recommendation to cite specific previously published works, please review and evaluate these publications to determine whether they are relevant and should be cited. There is no requirement to cite these works unless the editor has indicated otherwise.

Additional Editor Comments:

Please see below for additional comments from the academic editor for your careful consideration and appropriate response.

Thank you for your comprehensive responses to the comments provided by the three reviewers. Your revisions have strengthened both the quality and substance of the manuscript. Nevertheless, additional refinements are needed to further enhance its clarity, accuracy, and overall readiness for publication. Please find specific suggestions below.

Introduction

Although the introduction includes many of the essential components, its readability is affected by repetitions, structural gaps, and a few factual inaccuracies. For instance, the impacts of climate change are reiterated several times in the first two paragraphs, and the emergence and re emergence of diseases as climate related outcomes are mentioned in multiple places. Additionally, the statement in lines 86–87 that “health systems have a responsibility to maintain social determinants of health using a holistic approach” does not appear to be conceptually accurate.

To improve coherence and flow, I recommend fully restructuring the introduction section as follows:

• Paragraph 1: Define the health system, explaining its dimensions at both facility and community levels; describe its building blocks; introduce the concept of health system resilience in relation to climate change; and outline factors that support resilience.

• Paragraph 2: Define climate change and describe its potential impact and implications for health systems and their resilience.

• Paragraph 3: Review existing evidence on health system resilience to climate change, highlighting what is known and the evidence gaps this scoping review intends to address.

• Paragraph 4: State the research objectives and guiding questions.

Results

• The current PRISMA ScR diagram requires redrawing for better clarity and completeness, ensuring that all elements raised by Reviewer 2 are fully incorporated.

• Table 1 appears to be missing; you may consider relabeling the current Table 2 accordingly.

• Content in lines 275–279 should be moved to the Methods section, as the Results should focus exclusively on reporting outcomes of the quality assessment and findings.

Discussion

The discussion covers most of the expected components but would benefit from a more concise and structured presentation, as also previously recommended by Reviewer 2. I suggest reorganizing this section as follows:

• Paragraph 1: Summarize the study objectives and key findings, including the breadth of available literature, evidence related to the health system building blocks (governance and leadership, human resources, health information management, and service delivery), the limited evidence on health financing and essential medicines/health technologies, and the challenges identified.

• Paragraphs 2–4: Provide a detailed discussion of findings, exploring the factors that may explain observed trends and comparing them with results from other studies.

• Paragraph 5: Present the identified evidence gaps and discuss their implications for future research.

• Paragraph 6: Outline the study’s limitations.

[Note: HTML markup is below. Please do not edit.]

[NOTE: If reviewer comments were submitted as an attachment file, they will be attached to this email and accessible via the submission site. Please log into your account, locate the manuscript record, and check for the action link "View Attachments". If this link does not appear, there are no attachment files.]

To ensure your figures meet our technical requirements, please review our figure guidelines: https://journals.plos.org/plosone/s/figures

You may also use PLOS’s free figure tool, NAAS, to help you prepare publication quality figures: https://journals.plos.org/plosone/s/figures#loc-tools-for-figure-preparation.

NAAS will assess whether your figures meet our technical requirements by comparing each figure against our figure specifications.

# Author comment: Author response to Decision Letter 2

**DOI:** 10.1371/journal.pone.0349448.r004

17 Apr 2026

Dear Editor,

Greetings. I am pleased to submit the revised manuscript following your recommendations. I have attached a point-by-point response to what you raised in the recommendation letter. We have tried our best to make sure that the revised manuscript does not overlap with its protocol that was published in the same journal sometime back. I hope the current manuscript has improved because of your valuable recommendations. We look forward to your assessment and recommendation for the revised manuscript.

Receive my kind regards,

Chancy Chimatiro

Corresponding Authors

Attachment Submitted filename: RESPONSE TO EDITOR.docx

# Editor report: Decision Letter 2

**DOI:** 10.1371/journal.pone.0349448.r005

**Authors:** Olushayo Olu

24 Apr 2026

PONE-D-25-44483R2

Health system strategies and responses to the effects of climate change in Sub-Saharan Africa: A scoping review

PLOS One

Dear Dr. Chimatiro,

Thank you for submitting your manuscript to PLOS ONE. After careful consideration, we feel that it has merit but does not fully meet PLOS ONE’s publication criteria as it currently stands. Therefore, we invite you to submit a revised version of the manuscript that addresses the points raised during the review process.

Please submit your revised manuscript by Jun 08 2026 11:59PM. If you will need more time than this to complete your revisions, please reply to this message or contact the journal office at plosone@plos.org. When you're ready to submit your revision, log on to https://www.editorialmanager.com/pone/ and select the 'Submissions Needing Revision' folder to locate your manuscript file.

Please include the following items when submitting your revised manuscript:

- A letter that responds to each point raised by the academic editor and reviewer(s). You should upload this letter as a separate file labeled 'Response to Reviewers'.
- A marked-up copy of your manuscript that highlights changes made to the original version. You should upload this as a separate file labeled 'Revised Manuscript with Track Changes'.
- An unmarked version of your revised paper without tracked changes. You should upload this as a separate file labeled 'Manuscript'.

If you would like to make changes to your financial disclosure, please include your updated statement in your cover letter. Guidelines for resubmitting your figure files are available below the reviewer comments at the end of this letter.

If applicable, we recommend that you deposit your laboratory protocols in protocols.io to enhance the reproducibility of your results. Protocols.io assigns your protocol its own identifier (DOI) so that it can be cited independently in the future. For instructions see: https://journals.plos.org/plosone/s/submission-guidelines#loc-laboratory-protocols. Additionally, PLOS ONE offers an option for publishing peer-reviewed Lab Protocol articles, which describe protocols hosted on protocols.io. Read more information on sharing protocols at https://plos.org/protocols?utm_medium=editorial-email&utm_source=authorletters&utm_campaign=protocols.

As the corresponding author, your ORCID iD is verified in the submission system and will appear in the published article. PLOS supports the use of ORCID, and we encourage all coauthors to register for an ORCID iD and use it as well. Please encourage your coauthors to verify their ORCID iD within the submission system before final acceptance, as unverified ORCID iDs will not appear in the published article. *Only*  the individual author can complete the verification step; PLOS staff *cannot*  verify ORCID iDs on behalf of authors.

We look forward to receiving your revised manuscript.

Kind regards,

Olushayo Oluseun Olu

Academic Editor

PLOS One

Journal Requirements:

If the reviewer comments include a recommendation to cite specific previously published works, please review and evaluate these publications to determine whether they are relevant and should be cited. There is no requirement to cite these works unless the editor has indicated otherwise.

Please review your reference list to ensure that it is complete and correct. If you have cited papers that have been retracted, please include the rationale for doing so in the manuscript text, or remove these references and replace them with relevant current references. Any changes to the reference list should be mentioned in the rebuttal letter that accompanies your revised manuscript. If you need to cite a retracted article, indicate the article’s retracted status in the References list and also include a citation and full reference for the retraction notice.

Additional Editor Comments:

Many thanks for addressing the editorial comments provided during the last round of peer review. This has further improved the quality of your paper. Nevertheless, more work is required to improve the manuscript as follow:

1. Why are all tables and figures included as supplementary? At the least, the current supplementary figure 1 and supplementary table 3 should be included in the manuscript text.

2. No mention is made of supplementary tables 1-2 in the manuscript text? Where are they?

3. Lines 244-249 fits more in the methods section. You should then present the result of the MMAT in the result section.

4. In line 245, please correct the referencing style to be consistent with the rest of the document.

5. Please introduce a sub-heading in line 270 (just before service delivery) to read "Health system building blocks strategies for mitigating the impact of climate change"

6. Line 436-443 is unclear. Is this conclusion from the finding of your review or the finding of other studies? Please make this clearer

7. Further copy-editing and proof reading of the manuscript is required to correct some errors that may have been included in the use of track change mode.

Thank you

[Note: HTML markup is below. Please do not edit.]

To ensure your figures meet our technical requirements, please review our figure guidelines: https://journals.plos.org/plosone/s/figures

You may also use PLOS’s free figure tool, NAAS, to help you prepare publication quality figures: https://journals.plos.org/plosone/s/figures#loc-tools-for-figure-preparation.

NAAS will assess whether your figures meet our technical requirements by comparing each figure against our figure specifications.

# Author comment: Author response to Decision Letter 3

**DOI:** 10.1371/journal.pone.0349448.r006

29 Apr 2026

We are very grateful for the effective review given to our manuscript. We are looking forward to the outcome.

Attachment Submitted filename: RESPONSE TO EDITOR-2.docx

# Editor report: Decision Letter 3

**DOI:** 10.1371/journal.pone.0349448.r007

**Authors:** Olushayo Olu

1 May 2026

Health system strategies and responses to the effects of climate change in Sub-Saharan Africa: A scoping review

PONE-D-25-44483R3

Dear Dr. Chimatiro,

We’re pleased to inform you that your manuscript has been judged scientifically suitable for publication and will be formally accepted for publication once it meets all outstanding technical requirements.

Within one week, you’ll receive an e-mail detailing the required amendments. When these have been addressed, you’ll receive a formal acceptance letter and your manuscript will be scheduled for publication.

An invoice will be generated when your article is formally accepted. Please note, if your institution has a publishing partnership with PLOS and your article meets the relevant criteria, all or part of your publication costs will be covered. Please make sure your user information is up-to-date by logging into Editorial Manager at Editorial Manager® and clicking the ‘Update My Information' link at the top of the page. For questions related to billing, please contact billing support.

If your institution or institutions have a press office, please notify them about your upcoming paper to help maximize its impact. If they’ll be preparing press materials, please inform our press team as soon as possible -- no later than 48 hours after receiving the formal acceptance. Your manuscript will remain under strict press embargo until 2 pm Eastern Time on the date of publication. For more information, please contact onepress@plos.org.

Kind regards,

Olushayo Oluseun Olu

Academic Editor

PLOS One

Additional Editor Comments (optional):

Please ensure careful attention is given to the nomenclature of figures and tables prior to final publication. Supplementary tables and figures should be appropriately cited within the main manuscript but will be presented as annexes in the final published article. Accordingly, the labeling of supplementary and main tables should be clearly distinguished. For example, “Supplementary Tables 1, 2, 3,” versus “Tables 1, 2, 3.” What is the difference between supplementary table 3 and table 4? You may wish to retain only table 4 which should be included in the main article as table 1 as previously advised. Thus, your final manuscript will have supplementary tables and 1 and table 1. Thank you.

Reviewers' comments:

# Editor report: Acceptance letter

**DOI:** 10.1371/journal.pone.0349448.r008

**Authors:** Olushayo Olu

PONE-D-25-44483R3

PLOS One

Dear Dr. Chimatiro,

I'm pleased to inform you that your manuscript has been deemed suitable for publication in PLOS One. Congratulations! Your manuscript is now being handed over to our production team.

At this stage, our production department will prepare your paper for publication. This includes ensuring the following:

* All references, tables, and figures are properly cited

* All relevant supporting information is included in the manuscript submission,

* There are no issues that prevent the paper from being properly typeset

You will receive further instructions from the production team, including instructions on how to review your proof when it is ready. Please keep in mind that we are working through a large volume of accepted articles, so please give us a few days to review your paper and let you know the next and final steps.

Lastly, if your institution or institutions have a press office, please let them know about your upcoming paper now to help maximize its impact. If they'll be preparing press materials, please inform our press team within the next 48 hours. Your manuscript will remain under strict press embargo until 2 pm Eastern Time on the date of publication. For more information, please contact onepress@plos.org.

You will receive an invoice from PLOS for your publication fee after your manuscript has reached the completed accept phase. If you receive an email requesting payment before acceptance or for any other service, this may be a phishing scheme. Learn how to identify phishing emails and protect your accounts at https://explore.plos.org/phishing.

If we can help with anything else, please email us at customercare@plos.org.

Thank you for submitting your work to PLOS ONE and supporting open access.

Kind regards,

PLOS ONE Editorial Office Staff

on behalf of

Dr. Olushayo Oluseun Olu

Academic Editor

PLOS One
